# Supplementary material for: Early predictive factors of progression from severe type to critical ill type in patients with Coronavirus Disease 2019: A retrospective cohort study
Source: PLoS One. 2020 Dec 2;15(12):e0243195. doi: 10.1371/journal.pone.0243195 (PMC7710080; doi:10.1371/journal.pone.0243195)
Supplement: S1 Table — (DOCX) [file pone.0243195.s001.docx]

**S1 Table. Normal ranges of laboratory tests**

| **Variables** | **Normal range** |
| --- | --- |
| Full blood count |  |
| White blood cell, ×10^9^/L | 3.5-9.5 |
| Neutrophil, ×10^9^/L | 1.8-6.3 |
| Lymphocyte, ×10^9^/L | 1.1-3.2 |
| Hemoglobin, g/dL | 13.0-17.5 |
| Platelet, ×10^9^/L | 125-350 |
| Biochemical tests |  |
| Alanine aminotransferase, U/L | ≤41 |
| Aspartate aminotransferase, U/L | ≤40 |
| Albumin, g/L | 35-52 |
| Creatinine, μmol/L | 59-104 |
| Blood urea nitrogen, mmol/L | 3.6-9.5 |
| Lactate dehydrogenase, U/L | 135-225 |
| Myoglobin, ng/mL | ≤154.9 |
| Hypersensitive cardiac troponin I, pg/mL | ≤34.2 |
| Creatine kinase-MB, ng/mL | ≤7.2 |
| N-terminal pro-brain natriuretic peptide, pg/mL | <241 |
| Coagulation function |  |
| Prothrombin time, s | 11.5-14.5 |
| Activated partial thromboplastin time, s | 29.0-42.0 |
| D-dimer, μg/mL | <0.5 |
