# Supplementary material for: Early predictive factors of progression from severe type to critical ill type in patients with Coronavirus Disease 2019: A retrospective cohort study
Source: PLoS One. 2020 Dec 2;15(12):e0243195. doi: 10.1371/journal.pone.0243195 (PMC7710080; doi:10.1371/journal.pone.0243195)
Supplement: S2 Table — (DOCX) [file pone.0243195.s002.docx]

**S2 Table. Predictors of progression from severe type to critical type in COVID-19 patients (univariable logistic regression analysis)**

| Variables | All patients | | | Severe cases at admission | | |
| --- | --- | --- | --- | --- | --- | --- |
|  | Number [missing data] | Odds ratio (95% CI) | P value | Number [missing data] | Odds ratio (95% CI) | P value |
| Demographics |  |  |  |  |  |  |
| Age, years | 138 [0] | 1.035 (1.004-1.067) | **0.028** | 122 [0] | 1.027 (0.990-1.065) | 0.161 |
| Male gender | 71 [0] | 2.196 (0.989-4.879) | **0.053** | 61 [0] | 2.483 (0.876-7.037) | **0.087** |
| Symptoms since onset |  |  |  |  |  |  |
| Fever | 119 [0] | 1.060 (0.352-3.189) | 0.918 | 106 [0] | 0.748 (0.156-3.594) | 0.717 |
| Peak body temperature, °C | 115 [23] | 1.028 (0.579-1.827) | 0.924 | 103 [19] | 1.192 (0.587-2.422) | 0.627 |
| Cough | 116 [0] | 1.641 (0.515-5.229) | 0.402 | 102 [0] | 1.800 (0.382-8.488) | 0.458 |
| Dyspnea | 92 [0] | 2.437 (0.972-6.110) | **0.057** | 78 [0] | 1.706 (0.570-5.105) | 0.339 |
| Expectoration | 85 [1] | 2.105 (0.896-4.945) | **0.087** | 74 [1] | 2.733 (0.848-8.813) | **0.092** |
| Myalgia | 66 [1] | 1.022 (0.474-2.203) | 0.957 | 59 [1] | 1.202 (0.451-3.205) | 0.713 |
| Headache | 49 [0] | 1.295 (0.588-2.853) | 0.521 | 41 [0] | 0.897 (0.314-2.562) | 0.839 |
| Nausea | 47 [0] | 0.714 (0.309-1.647) | 0.429 | 43 [0] | 0.823 (0.289-2.347) | 0.716 |
| Palpitation | 36 [1] | 1.412 (0.607-3.284) | 0.424 | 31 [1] | 1.422 (0.489-4.133) | 0.518 |
| Night sweats | 32 [1] | 0.770 (0.300-1.977) | 0.587 | 29 [1] | 0.821 (0.249-2.704) | 0.746 |
| Sore throat | 30 [0] | 0.520 (0.182-1.484) | 0.222 | 29 [0] | 0.832 (0.253-2.739) | 0.762 |
| Vomiting | 29 [0] | 0.920 (0.355-2.387) | 0.865 | 25 [0] | 0.690 (0.184-2.584) | 0.582 |
| Chest pain | 26 [0] | 1.105 (0.421-2.905) | 0.839 | 22 [0] | 0.829 (0.219-3.134) | 0.782 |
| Hemoptysis | 18 [0] | 2.091 (0.741-5.902) | 0.164 | 14 [0] | 1.568 (0.394-6.250) | 0.524 |
| Comorbidities |  |  |  |  |  |  |
| Hypertension | 63 [0] | 1.170 (0.543-2.523) | 0.688 | 53 [0] | 0.723 (0.263-1.984) | 0.529 |
| Diabetes | 28 [0] | 1.230 (0.486-3.110) | 0.662 | 27 [0] | 2.421 (0.845-6.934) | **0.100** |
| Coronary artery disease | 26 [0] | 0.651 (0.225-1.881) | 0.428 | 24 [0] | 0.732 (0.195-2.749) | 0.644 |
| Pulmonary diseases *^a^* | 21 [0] | 2.051 (0.770-5.466) | 0.151 | 16 [0] | 1.298 (0.332-5.075) | 0.708 |
| Chronic kidney diseases *^b^* | 9 [0] | 2.529 (0.639-10.007) | 0.186 | 7 [0] | 2.306 (0.413-12.862) | 0.341 |
| Smoking history | 29 [8] | 2.328 (0.955-5.674) | **0.063** | 23 [7] | 1.852 (0.579-5.918) | 0.299 |
| From onset to admission, days | 138 [0] | 1.021 (0.973-1.071) | 0.400 | 122 [0] | 1.031 (0.975-1.089) | 0.286 |
| From diagnosis to admission, days | 131 [7] | 1.047 (0.992-1.106) | **0.098** | 116 [6] | 1.066 (1.001-1.136) | **0.048** |
| Treatments before admission |  |  |  |  |  |  |
| Antiviral treatment *^c^* | 98 [0] | 2.772 (1.237-6.211) | **0.013** | 91 [0] | 1.920 (0.680-5.422) | 0.218 |
| Arbidol | 38 [1] | 0.188 (0.054-0.658) | **0.009** | 37 [0] | 0.229 (0.050-1.046) | **0.057** |
| Oseltamivir | 46 [1] | 0.642 (0.271-1.520) | 0.314 | 43 [0] | 0.823 (0.289-2.347) | 0.716 |
| Antibiotics *^d^* | 94 [0] | 2.256 (1.019-4.991) | **0.045** | 86 [0] | 1.948 (0.710-5.343) | 0.195 |
| Fluoroquinolones | 67 [0] | 0.385 (0.171-0.867) | **0.021** | 64 [0] | 0.610 (0.227-1.642) | 0.328 |
| β-lactams | 22 [0] | 1.467 (0.543-3.958) | 0.450 | 18 [0] | 1.100 (0.285-4.240) | 0.890 |
| Nonsteroidal anti-inflammatory drugs | 16 [0] | 0.385 (0.083-1.787) | 0.223 | 16 [0] | 0.748 (0.156-3.594) | 0.717 |
| Glucocorticoids | 16 [0] | 1.394 (0.448-4.335) | 0.566 | 14 [0] | 1.568 (0.394-6.250) | 0.524 |
| Gamma globulin | 12 [3] | 1.550 (0.436-5.513) | 0.498 | 10 [3] | 1.453 (0.283-7.474) | 0.655 |
| Vital signs at admission |  |  |  |  |  |  |
| Heart rate, bpm | 133 [5] | 1.022 (1.000-1.044) | **0.049** | 117 [5] | 1.019 (0.989-1.051) | 0.211 |
| Systolic blood pressure, mmHg | 124 [14] | 0.999 (0.981-1.017) | 0.913 | 108 [14] | 0.988 (0.963-1.013) | 0.328 |
| Diastolic blood pressure, mmHg | 121 [17] | 1.000 (0.972-1.028) | 0.985 | 105 [17] | 0.988 (0.951-1.026) | 0.527 |
| Respiratory rate, bpm | 102 [36] | 1.057 (1.003-1.115) | **0.039** | 88 [34] | 1.028 (0.978-1.080) | 0.281 |
| Pulse oxygen saturation <93% | 48 [1] | 8.587 (3.605-20.456) | **<0.001** | 37 [1] | 9.617 (3.132-29.528) | **<0.001** |
| Full blood count at admission |  |  |  |  |  |  |
| White blood cell, ×10^9^/L | 138 [0] | 1.503 (1.288-1.755) | **<0.001** | 122 [0] | 1.403 (1.167-1.686) | **<0.001** |
| Neutrophil, ×10^9^/L | 138 [0] | 1.626 (1.359-1.946) | **<0.001** | 122 [0] | 1.491 (1.222-1.820) | **<0.001** |
| Lymphocyte, ×10^9^/L | 138 [0] | 0.199 (0.076-0.521) | **0.001** | 122 [0] | 0.302 (0.098-0.932) | **0.037** |
| Hemoglobin, g/dL | 138 [0] | 1.077 (0.886-1.309) | 0.459 | 122 [0] | 1.057 (0.798-1.400) | 0.701 |
| Platelet, ×10^9^/L | 138 [0] | 0.998 (0.994-1.002) | 0.345 | 122 [0] | 0.997 (0.991-1.002) | 0.267 |
| Biochemical tests at admission |  |  |  |  |  |  |
| Alanine aminotransferase, U/L | 138 [0] | 1.002 (0.992-1.011) | 0.735 | 122 [0] | 1.000 (0.988-1.013) | 0.968 |
| Aspartate aminotransferase, U/L | 138 [0] | 1.017 (1.004-1.030) | **0.013** | 122 [0] | 1.013 (0.996-1.030) | 0.141 |
| Albumin, g/L | 138 [0] | 0.859 (0.781-0.944) | **0.002** | 122 [0] | 0.916 (0.823-1.021) | 0.112 |
| Creatinine, μmol/L | 138 [0] | 1.001 (0.999-1.004) | 0.329 | 122 [0] | 1.000 (0.997-1.004) | 0.889 |
| Blood urea nitrogen, mmol/L | 138 [0] | 1.370 (1.188-1.579) | **<0.001** | 122 [0] | 1.255 (1.068-1.476) | **0.006** |
| Lactate dehydrogenase, U/L | 138 [0] | 1.008 (1.005-1.011) | **<0.001** | 122 [0] | 1.006 (1.003-1.009) | **0.001** |
| Myoglobin, ng/mL | 136 [2] | 1.005 (1.002-1.008) | **0.003** | 121[1] | 1.003 (1.001-1.006) | **0.012** |
| Hypersensitive cTnI, pg/mL | 137 [1] | 1.006 (1.000-1.012) | **0.067** | 122 [0] | 1.001 (0.995-1.008) | 0.642 |
| Creatine kinase-MB, ng/mL | 136 [2] | 2.662 (1.671-4.242) | **<0.001** | 121[1] | 2.005 (1.243-3.235) | **0.004** |
| NT-proBNP, pg/mL | 136 [2] | 1.000 (1.000-1.000) | 0.136 | 121[1] | 1.000 (1.000-1.000) | 0.413 |
| Coagulation function at admission |  |  |  |  |  |  |
| Prothrombin time, s | 138 [0] | 2.848 (1.804-4.495) | **<0.001** | 122 [0] | 1.994 (1.242-3.203) | **0.004** |
| Activated partial thromboplastin time, s | 138 [0] | 1.062 (1.006-1.121) | **0.028** | 122 [0] | 1.094 (1.024-1.169) | **0.008** |
| D-dimer, μg/mL | 138 [0] | 1.186 (1.090-1.290) | **<0.001** | 122 [0] | 1.101 (0.998-1.215) | **0.054** |

cTnI, cardiac troponin I; NT-proBNP, N-terminal pro-brain natriuretic peptide. Numbers in square brackets indicate patients with missing data.

*^a^* Includes asthma, chronic obstructive pulmonary disease, and interstitial lung disease.

*^b^* Defined as glomerular filtration rate (GFR) <60 mL/min per 1·73 m² or markers of kidney damage, or both, of at least 3 months duration.

*^c^* Includes arbidol, oseltamivir, lopinavir/ritonavir, interferon, ganciclovir, and ribavirin.

*^d^* Includes fluoroquinolones, β-lactams, and macrolides.
