# Supplementary material for: Early predictive factors of progression from severe type to critical ill type in patients with Coronavirus Disease 2019: A retrospective cohort study
Source: PLoS One. 2020 Dec 2;15(12):e0243195. doi: 10.1371/journal.pone.0243195 (PMC7710080; doi:10.1371/journal.pone.0243195)
Supplement: Attachment — Submitted filename: R2-Response to Reviewers.docx [file pone.0243195.s004.docx]

**Response to Reviewers**

Journal Requirements:

1. Please ensure that your manuscript meets PLOS ONE's style requirements, including those for file naming.

Response: Thank you for reminding us. We have confirmed that our manuscript met PLOS ONE's style requirements, including those for file naming.

2. In ethics statement in the manuscript and in the online submission form, please provide additional information about the patient records/samples used in your retrospective study. Specifically, please ensure that you have discussed whether all data were fully anonymized before you accessed them and/or whether the IRB or ethics committee specifically waived the requirement for informed consent.

Response: Thank you. We have provided additional information about the patient records used in our retrospective study. “Considering that the study was retrospective in nature and no follow-up was performed, the Ethics Committee agreed to waive written informed consent. Personal data of patients were kept strictly confidential.” (page 6, lines 112-114).

3. We note that you have indicated that data from this study are available upon request. PLOS only allows data to be available upon request if there are legal or ethical restrictions on sharing data publicly.

Response: Thank you. We have uploaded the minimal anonymized data set necessary to replicate our study findings as Supporting Information file (S3 Dataset: Relevant data underlying the main results.).

Reviewer #2:

1. The English language needs to be revised for style.

Response: Thank you. We have revised the English language style.
